# Reciprocal Roles of Angiotensin II and Angiotensin II Receptors Blockade (ARB) in Regulating Cbfa1/RANKL via cAMP Signaling Pathway: Possible Mechanism for Hypertension-Related Osteoporosis and Antagonistic Effect of ARB on Hypertension-Related Osteoporosis

**DOI:** 10.3390/ijms12074206

**Published:** 2011-06-27

**Authors:** Xiao-Xu Guan, Yi Zhou, Ji-Yao Li

**Affiliations:** 1 State Key Laboratory of Oral Diseases, Sichuan University, Chengdu 610041, China; E-Mails: gsunrise@yahoo.cn (X.-X.G.); zhouyizyzyzy@163.com (Y.Z.); 2 West China Hospital of Stomatology, Sichuan University, Chengdu 610041, China

**Keywords:** hypertension, osteoporosis, angiotensin II, ARB, Cbfa1, RANKL, cAMP

## Abstract

Hypertension is a risk factor for osteoporosis. Animal and epidemiological studies demonstrate that high blood pressure is associated with increased calcium loss, elevated parathyroid hormone, and increased calcium movement from bone. However, the mechanism responsible for hypertension-related osteoporosis remains elusive. Recent epidemiological studies indicate the benefits of Angiotensin II Receptors Blockade (ARB) on decreasing fracture risks. Since receptors for angiotensin II, the targets of ARB, are expressed in both osteoblasts and osteoclasts, we postulated that angiotensin II plays an important role in hypertension-related osteoporosis. Cbfa1 and RANKL, the important factors for maintaining bone homeostasis and key mediators in controlling osteoblast and osteoclast differentiation, are both regulated by cAMP-dependent signaling. Angiotensin II along with factors such as LDL, HDL, NO and homocysteine that are commonly altered both in hypertension and osteoporosis, can down-regulate the expression of Cbfa1 but up-regulate RANKL expression via the cAMP signaling pathway. We thus hypothesized that, by altering the ratio of Cbfa1/RANKL expression via the cAMP-dependent pathway, angiotensin II differently regulates osteoblast and osteoclast differentiation leading to enhanced bone resorption and reduced bone formation. Since ARB can antagonize the adverse effect of angiotensin II on bone by lowering cAMP levels and modifying other downstream targets, including LDL, HDL, NO and Cbfa1/RANKL, we propose the hypothesis that the antagonistic effects of ARB may also be exerted via cAMP signaling pathway.

## 1. Introduction

Osteoporosis is a common disorder that leads to reduction in bone mass, deterioration in bone microarchitecture, susceptibility to skeletal fragility, and increased risk of fracture [1]. In addition to physiological reasons like age and estrogen deficiency, pathological conditions such as multiple myelomatosis, hyperthyroidism, diabetes, and hypertension are also proved to be related to osteoporosis [2–4].

Osteoporosis and hypertension are two serious public health issues among the elderly population. Hypertension is a risk factor for osteoporosis [4–6]. Animal and epidemiological studies have shown that high blood pressure is associated with abnormal calcium metabolism, including an increase in urinary calcium excretion, a raised parathyroid hormone level and a tendency for low serum ionized calcium levels [5,6]. This calcium leakage could lead to increased movement of calcium from bone and thereby increase the risk for osteoporosis. The strategies now used to manage osteoporosis include selective estrogen receptor modulators, hormone replacement therapy, calcitonin and bisphosphonates [2]. Although these pharmacologic therapies are available, they have no effect on hypertension-related osteoporosis.

Recent epidemiological studies, however, reported the benefit of antihypertensive drugs, such as angiotensin-converting enzyme inhibitors (ACEI) and ARB, on increasing bone mass and decreasing the risk for bone fractures [7–11]. Since the main physiological function of these substances is blocking the activation of receptors for angiotensin II, we hypothesized that the renin-angiotensin system (RAS), from which angiotensin II is synthesized, might be involved in bone metabolism [7,8]. Moreover, based on the expression of receptors for angiotensin II in both osteoblasts and osteoclasts [9,12], we postulated that angiotensin II may influence osteoblast and osteoclast differentiation, and then cause the imbalance between bone formation and bone absorption. We also inferred that the negative impact of angiotensin II on bone may be antagonized by ARB.

## 2. Angiotensin II Regulates Cbfa1/RANKL via the cAMP Signaling Pathway: The Possible Mechanism for Hypertension-Related Osteoporosis

One well-known mechanism involved in bone homeostasis is that the resistance and integrity of bone depends upon the balance between bone formation by osteoblasts and bone resorption by osteoclasts [2,13–15]. Thus, any factor that decreases osteoblast number and their ability to form bone or increase osteoclast number and their ability to induce bone absorption may trigger or accelerate the process of osteoporosis. The differentiation of osteoblast and osteoclasts is primarily controlled by two key mediators, Cbfa1 and RANKL, which are regulated by the second messenger, cAMP [13–15].

It has been demonstrated by several studies that pathological conditions, including abnormal lipid metabolism, altered levels of low density lipoprotein (LDL), high density lipoprotein (HDL), plasma homocysteine and nitric oxide (NO), are involved both in hypertension and osteoporosis [16–20]. Elevated LDL, increased plasma homocysteine, and low HDL were associated with reduced bone mineral density and are risk factors for osteoporosis [17–19]. Although NO is a key inflammatory mediator and causes bone absorption in the case of inflammation [20], NO has a beneficial effect on bone due to its ability to increase bone formation via stimulating osteoblast proliferation and differentiation, and regulating postnatal bone formation [21,22].

Interestingly, the administration of angiotensin II is able to modify these molecules. Angiotensin II can enhance vascular endothelial cells (VECs) from coronary artery and umbilical vein to take up LDL by up regulating LDL endothelial receptors; suppress HDL-induced endothelial NO synthase via hSR-BI/CLA-1 in VECs; impair the insulin-induced production of NO via IRS-1/PI3-kinase/Akt/eNOS pathway in VECs from umbilical vein [23–27]. However, all these actions of angiotensin II impair bone formation and may contribute to accelerate the process of osteoporosis [17–21].

cAMP is a key intracellular signaling molecule in controlling bone homeostasis [13,15]. Interestingly, the levels of cAMP in plasma and urine were elevated both in osteoporotic and hypertensive patients [28,29]. Therefore, there is a possibility that in hypertensive osteoporotic patients, the levels of cAMP in plasma and urine could be much higher and that the abnormal cAMP signaling pathway may contribute to hypertension-related osteoporosis.

The activation of cAMP signaling plays an important role in mediating the levels of intracellular HDL in aortic VECs [30]. cAMP is also an essential mediator for the pathological roles of homocysteine and LDL in the induction of endothelial dysfunction and damage, and VECs’ ability to produce NO [31–34]. Angiotensin II is capable of stimulating an increase of intracellular cAMP [35] and then activating downstream signaling pathways that are involved in the regulation of LDL, homocysteine and NO, which in turn modifies Cbfa1 expression [36–38]. It is thus plausible that angiotensin II alters the expression of Cbfa1 and subsequently reduces osteoblast number to cause impaired bone formation by activating the cAMP signaling pathway and regulating other downstream targets (LDL, homocysteine and NO). Moreover, angiotensin II also shows the ability to inhibit the expression of osteocalcin and decrease the activity of alkaline phosphatase, both of which are essential for bone matrix synthesis and maturation, and regulated by the Cbfa1 promoter [39,40].

In contrast to the role of angiotensin II on Cbfa1, the expression of RANKL in osteoblasts, a marker for osteoclastic activation, is significantly increased by angiotensin II [12]. Additionally, elevated LDL, increased plasma homocysteine, low levels of HDL and NO that are common features of patients with both hypertension and osteoporosis, are also in favor of promoting RANKL expression and osteoclast activity [41–44]. Since RANKL is also mediated by cAMP-dependent signaling [13,14], we hypothesized that angiotensin II alters the ratio of Cbfa1/RANKL expression via the cAMP signaling pathway, subsequently increases the number of osteoclasts at the expense of osteoblast, and eventually causes the imbalance between bone formation and bone absorption.

## 3. Inverse Effects of ARB on Regulating Cbfa1/RANKL via cAMP Signaling Pathway: The Possible Mechanism for the Antagonistic Effect of ARB on Hypertension-Related Osteoporosis

In contrast to roles of angiotensin II on bone, there has been plentiful evidence from epidemiology, which testified that ARB shows the capability to decrease the risk of fractures but increases bone mass [7–11]. Moreover, when compared to the favorable role of angiotensin II on osteoclasts, ARB treatment can inversely influence osteoclastic activity by abolishing the angiotensin II–induced up regulation of RANKL expression in osteoblasts [12]. ARB also exhibits abilities to enhance Cbfa1-induced osteoblast differentiation [45]. Therefore, considering the completely opposite roles of angiotensin II and ARB on bone and on regulating bone-related key genes Cbfa1/RANKL, we put forward a hypothesis that a common pathway may exist and be shared by angiotensin II and ARB to change the ratio of Cbfa1/RANKL, and then regulate bone metabolism inversely.

Interestingly, in comparison to the role of angiotensin II in regulating LDL, HDL and NO [23–27], ARB plays a totally different role on these key molecules in VECs or in the plasma of hypertension patients except homocysteine [24,46–48]. Given that these molecules are regulated by the cAMP signaling pathway, we hypothesized that ARB may reverse the deleterious effects of angiotensin II on bone by cAMP signaling pathway. Indeed, the angiotensin II induced-migration of coronary artery medial smooth muscle cells via the cAMP signaling pathway can be blocked by ARB, losartan [49]. It seems that ARB has the ability to temper the biological effects of cAMP possibly via decreasing the level of cAMP. Therefore, these data suggested a possibility that ARB can also regulate the cAMP signaling pathway. By differently regulating the cAMP signaling pathway, ARB may reverse the ratio of Cbfa1/RANKL gene expression, maintain the balance between bone formation and bone resorption, and antagonize the unfavorable effects of angiotensin II on bone.

## 4. Conclusions and Implications

Hypertension is a risk factor for osteoporosis. However, the mechanism underlying the increased risk of hypertension on osteoporosis is yet to be defined. We put forward a hypothesis from epidemiological studies that angiotensin II may play an important role in hypertension-related osteoporosis and ARB may antagonize the effect of angiotensin II on bone. The inverse roles of angiotensin II and ARB on bone may be through differently regulating bone-related key genes Cbfa1/RANKL via cAMP signaling pathway. ARB may also improve angiotensin II-induced abnormalities in the levels of LDL, HDL, NO and homocysteine that are common features of hypertension and osteoporosis. If our hypotheses are testified to be true, it may provide a possible molecular mechanism for hypertension-related osteoporosis.
